# On the Structural Plasticity of the Human Genome: Chromosomal Inversions Revisited

**DOI:** 10.2174/138920212803759703

**Published:** 2012-12

**Authors:** Joao M Alves, Alexandra M Lopes, Lounès Chikhi, António Amorim

**Affiliations:** 1 Doctoral Program in Areas of Basic and Applied Biology (GABBA), University of Porto, Portugal; 2 IPATIMUP - Instituto de Patologia e Imunologia Molecular da Universidade do Porto, Porto, Portugal; 3 Instituto Gulbenkian de Ciência (IGC), Oeiras, Portugal; 4 CNRS (Centre National de la Recherche Scientifique), Université Paul Sabatier, Ecole Nationale de Formation Agronomique, Unité Mixte de Recherche 5174 EDB (Laboratoire Évolution & Diversité Biologique), F-31062 Toulouse, France; 5 Faculdade de Ciências da Universidade do Porto, Porto, Portugal

**Keywords:** Chromosomal inversions, Genome architecture, Demographic history, Human evolution, Non-allelic homologous recombination, Segmental duplications.

## Abstract

With the aid of novel and powerful molecular biology techniques, recent years have witnessed a dramatic increase in the number of studies reporting the involvement of complex structural variants in several genomic disorders. In fact, with the discovery of Copy Number Variants (CNVs) and other forms of unbalanced structural variation, much attention has been directed to the detection and characterization of such rearrangements, as well as the identification of the mechanisms involved in their formation. However, it has long been appreciated that chromosomes can undergo other forms of structural changes - balanced rearrangements - that do not involve quantitative variation of genetic material. Indeed, a particular subtype of balanced rearrangement – inversions – was recently found to be far more common than had been predicted from traditional cytogenetics. Chromosomal inversions alter the orientation of a specific genomic sequence and, unless involving breaks in coding or regulatory regions (and, disregarding complex *trans* effects, in their close vicinity), appear to be phenotypically silent. Such a surprising finding, which is difficult to reconcile with the classical interpretation of inversions as a mechanism causing subfertility (and ultimately reproductive isolation), motivated a new series of theoretical and empirical studies dedicated to understand their role in human genome evolution and to explore their possible association to complex genetic disorders. With this review, we attempt to describe the latest methodological improvements to inversions detection at a genome wide level, while exploring some of the possible implications of inversion rearrangements on the evolution of the human genome.

## INTRODUCTION

Over the last years, a growing number of geneticists and evolutionary biologists are shifting their attention from single nucleotide polymorphisms (SNPs) towards bigger and more complex alterations in the architecture of eukaryotic genomes thus going back to some of the oldest genetic markers (e.g. [1, 2]). With the aid of novel and powerful molecular biology techniques (e.g. high-throughput sequencing platforms, array-Comparative Genomic Hybridization and SNP microarrays (see [3] for a review)), the study of the structural plasticity of the genome has gained momentum. Indeed, we are currently witnessing major advances in the field of molecular and computational genomics with increasingly high quality whole-genome data accumulating for several species and fast improvements in computational and statistical tools that allow the extraction of reliable information from these sources.

This has led to the discovery, validation and characterization of a whole set of different types of structural variants (SVs) and it is now evident that genomic variation is far more complex than previously thought [4]. SVs can be defined as a wide variety of balanced and unbalanced genomic rearrangements of different sizes. They range from Copy Number Variants (CNVs) such as insertions, deletions, and duplications, all being unbalanced, to chromosomal inversions (balanced) and translocations (unbalanced or balanced). Biomedical and clinically oriented research became particularly focused in genomic imbalances, and architectural changes, with genome-wide association studies (GWAS) regularly highlighting the involvement of SVs in several genomic disorders [5-7].

At present, much attention is being directed to the identification of the mechanisms and processes involved in their formation, however uncertainty remains regarding the contribution of these heteromorphisms to phenotype differences between individuals, since most variants described have been found in healthy individuals [8-10].

In this review we consider a particular subtype of rearrangement – chromosomal inversions – that has been increasingly recognized as a relatively common source of variation contrary to early predictions from classical cytogenetics [1]. 

Inversions alter the orientation of a specific genomic sequence and, for decades, they have been interpreted as a potential mechanical cause of subfertility (and ultimately reproductive isolation) since cross-over events (i.e. recombination) between inverted and non-inverted segments could result in unbalanced, and generally abortive, gametes [11, 12]. From an evolutionary point of view, inversions became recognized as privileged systems to study major processes (e.g. selection) [13] under the generalist idea often held but not always well defined that they could protect chromosomal regions from gene flow, and therefore act as an initial step towards genomic divergence [14]. Indeed, studies in chromosomal evolution have repeatedly attributed important evolutionary roles to these structural rearrangements, with several lines of evidence suggesting their involvement in phenotypic variability [15, 16], adaptive divergence within species [17, 18], and in the origin and evolution of sex chromosomes in mammals [1]. 

In humans, however, the role of inversions in disease or genome evolution remains unclear [8, 19]. At this stage, more than 1000 inversions have been deposited in the Database of Genomic Variants [20], involving all 22 autosomes, but the fact that only two inversion polymorphisms have been fully characterized at the population level [21-24] clearly illustrates the necessity of studying inversion polymorphisms at a larger scale [19].

Here, our aim is to review the latest theoretical and empirical work dedicated to chromosomal inversions in the human genome, either as disease-associated variants or as segregating polymorphisms in human populations. We discuss the recent advances made in the structural and genetic characterization of inversion polymorphisms - highlighting the major drawbacks in the current strategies - and important issues that have as yet received little attention. Furthermore, we will explore some of the evolutionary and demographic scenarios that have been invoked to explain the presence, maintenance and apparent rise in frequency of particular inversions in different human populations. 

## INVERSIONS – ON THE DETECTION OF BALANCED STRUCTURAL VARIANTS

The detection of inversions was traditionally limited to large-scale microscopically visible rearrangements via karyotype analysis using classical G-banding techniques [25-27]. With the implementation of improved comparative genomic strategies, both at population and species level, an extraordinary amount of previously unknown inversions were identified in recent years [28, 29]. While most experimental techniques (e.g. FISH, PGFE, Fusion-PCR) remain laborious and target-based [30], where one can only test the presence of a predicted inversion in a specific genomic location, new computational approaches have been recently introduced to identify or predict the location of inversions, from SNP array data and next-generation sequencing (NGS) data, at a genome-wide level [31-34].

For example, Bansal *et al.* [31] developed a statistical method to detect large polymorphic chromosomal segments (> 200 Kb) that are inverted in the majority of the chromosomes in a population, with respect to the human reference sequence and applied it to HapMap data. Even with limited statistical power to detect polymorphisms at frequencies lower than 0.25 (with respect to the human reference), a list of 176 candidate inversions was generated using this model, which overlapped with several previously known inversion polymorphisms. However, since the model uses patterns of strong, long range linkage disequilibrium (LD) to access putative sites of inversion rearrangements, some predicted inversions might be artifacts and may just represent regions of high LD due to low recombination or recent selective sweeps, as noted by the authors.

More recently, Sindi & Raphael [32] applied a probabilistic model, using differences in haplotype block structure, to identify inversion polymorphisms from this type of data. In opposition to [31], their method was able to predict inversion frequencies and detect inversions that are the minor allele in the population (i.e. where most individuals had the reference “non-inverted” haplotype). Furthermore, they generated a set of 355 putative inversion polymorphisms using SNP data from 4 populations (CEU, YRI, CHB+JPT), overlapping with several inversion polymorphisms that have already been validated by others, or for which direct evidence exists [3].

While it was possible to identify known inversion polymorphisms in both studies, hence validating the methods used, there are still several limitations that need to be considered when predicting inversion rearrangements from SNP-haplotype data. The proposed computational models rely on the assumption that (i) SNP haplotypes can be used as a proxy of the inversion status and (ii) strong LD is expected in regions harboring inversion rearrangements. As a consequence, only ancient inversions which have accumulated divergent mutations are likely to be captured. Another issue is that both models implicitly assume a single origin but multiple independent events might have given rise to the presence of a given inversion in different haplotypic backgrounds. Indeed, in the attempt to characterize 6 human disease-associated inversion polymorphisms, Antonacci *et al.* [19] showed, with the exception of one inversion (i.e. 17q21.31), no remarkable correlation between SNP-based haplotypes and the inversion structure. The authors concluded that each of these inversions may have occurred multiple times in the human lineage, on different haplotype backgrounds, providing evidence of recurrence. Similar results were later observed for the 17q21.31 inversion, in a cytogenetically-based study [35], where some individuals homozygous for the SNP-defined haplotype, previously thought to be completely associated with the inversion (i.e. H2), were in fact heterokaryotic (i.e. inversion heterozygotes). In the latter case, it shows that the “inverted haplo-type” is sometimes oriented like the “non-inverted” haplotype.

In summary, identifying inversions by means of high density SNP data is promising but far from a trivial task, and in those cases where an inversion has arisen independently on at least 2 distinct haplotype backgrounds, genotyping methods based on SNP data are prone to artifacts (e.g. false negatives). 

Alternatively, sequence-based computational approaches have been recently introduced to detect SVs (including inversions) making use of specific sequence data signatures (see [3]). Among others, paired-end mapping (PEM) algorithms are showing promising results in genome wide detection of inversion rearrangements as they are able to assess the orientation of paired-end reads, therefore allowing the identification of discordant mapping to a reference genome [36, 37]. A series of recent publications [33, 34, 38] have applied this new method to identify structural variants in the human genome, and while 56 inversions were found using a single individual [33], Kidd *et al.* [34] analyzing 8 genomes, identified a total of 217 inversions (but see [8] for a more comprehensive review). 

Considerable technological improvements have boosted our ability to assay inversion variants in the human genome. NGS is becoming a routinely used tool in many biological fields [39-43], and has already contributed (and is still contributing) to a better understanding of the architecture of the human genome. Nevertheless, such technologies still represent a challenge to present-day research [39, 44]. For instance, inversion breakpoints are generally enriched in runs of duplicated segments of DNA (e.g. segmental duplications (SDs)), which greatly limits the ability to unambiguously map breakpoint regions [29]. Also, upon discovery, independent validation methods are still required to confirm the orientation of a specific chromosome segment. 

Ultimately, validation studies that simultaneously take into account the limitations of the computational and molecular tools and experimental procedures are crucially needed to estimate the error rates of SNP- or NGS- inferred inversion rearrangements. Indeed, a recent review [44] explored the main limitations of the current approaches to discovering structural variants, highlighting the importance of designing algorithms that incorporate multiple methodologies to improve power, robustness, sensitivity and specificity. 

## INVERSIONS – ON THEIR IMPACT OF GENOME EVOLUTION

### Molecular Effects of Inversions

As balanced rearrangements, inversions do not involve quantitative alteration in the content of cellular DNA (at least no significant change in theory), but the reorganization of a genomic segment induces an alteration of the original genetic background which may have several repercussions. Although much uncertainty remains regarding the direct effects of inversions at the molecular level (e.g. gene expression patterns), it has been shown that some inversions can result in major phenotypic alterations. For instance, the split of the mammalian *Hoxd* gene cluster into two independent pieces, using an experimental technique (STRING) that induced an inversion rearrangement [45], was responsible for the loss of expression of *Hoxd *genes during limb development. One likely explanation to this observation is that the artificial repositioning of the genes within the inverted region, relatively to flanking regulatory elements, led to changes in patterns of gene activity [29, 46]. Inversions exert some of their effects indirectly, by imposing new regimens of molecular evolution on the DNA sequences encompassed by them. This is due to a reduction, or even suppression of recombination within these segments in *heterokaryotypes*. As subtle as it may seem, such effect can have drastic consequences since, by acting as a genetic barrier, an inversion may “freeze” an alternative allelic/haplotypic sequence in a population [47]. 

Indeed, ever since their first identification in the 1920s [48], inversions have been particularly investigated for their putative role in population divergence and speciation phenomena [14, 49-52]. While classic models (e.g. hybrid dysfunction model of speciation such as the Bateson-Dobzhansky-Muller [53]) often rely on the idea of fertility cost to hybrids, overlooking the mechanisms by which rearrangements become established in the first place, new inversion-based speciation models [14, 49-51] have been proposed in recent years invoking the suppression of recombination as a major process for genetic diversification and speciation.

Recombination is regarded as one of the major evolutionary processes since it is responsible for the genetic shuffling and introduction of new allelic combinations, upon which selection can act [52]. Once an inversion arises in a population, recombination in that region becomes suppressed between chromosomes with different orientations (with the exception of double cross-overs within large inverted regions). Virtually all “suppressed-recombination” models explicitly suggest that such rearrangements provide a window of opportunity for the accumulation of differences between the two chromosomal configurations that could culminate in the evolution of reproductive isolation [14, 49-52].

At present, observations supporting these new models have been reported for several species, including birds [54], mammals [55], insects [15] and plants [17]. In primates much controversy has been building up in the last years over the contribution of suppressed recombination to the divergence of ancestral populations of humans and chimpanzees and, in spite of many efforts, accelerated evolution in rearranged versus collinear chromosomes between the two species has not been definitely proven [56-58]. However, since the scope of this review falls exclusively on human polymorphic inversions, we will not explore further the role of inversions on speciation, but instead we will focus on the possible mechanisms and processes by which inversions rise in frequency and may become established in populations.

### From Genomic Novelties to Established Polymorphisms

The spread of these rearrangements can result from a combination of several factors, largely influenced by populations’ demography, ecology and evolutionary history. It has been argued [18, 49] that an inversion could rise in frequency because it brings together locally adapted genes that become “protected” from introgression, due to a local reduction in recombination. According to this scenario, the selective advantage is not directly related to the new chromosomal structure but to its favorable genetic (i.e. haplotypic) composition [59]. As a consequence, the distribution of such inversions may display clines related with local adaptation [18]. Non-ecological processes, such as meiotic drive (i.e. a process in which an allele is over-transmitted in gametes during meiosis), might also influence the frequency and distribution of an inversion polymorphism by distorting its segregation [1]. However, while this is theoretically possible such processes do not appear to be general features in establishing inversions in human populations, since most rearrangements seem to segregate normally. 

As any other type of mutation, inversions are affected by evolutionary forces. On this basis, random genetic drift, selection and gene flow (i.e. migration) can play major roles in shaping their distribution and frequencies across populations. For example, Spirito *et al.* [60], using a multi-deme model of local extinction and recolonization, observed that even underdominant inversions could, by chance, persist or rise to fixation in populations. However, the authors noted that this scenario is only achieved in cases of small effective population size, where drift causes the maintenance or rise in frequency of the rearrangement albeit the systematic pressure of selection. In contrast, if the rearrangement offers a selective advantage to the carriers, its fixation is more likely, due to the expected advantage of the inversion homozygotes [52, 59, 60].

In humans, numerous inversion variants of different sizes segregate in populations [8, 31, 46]. Although the vast majority falls within the 10 to 100kb size interval, there are several inversion polymorphisms with sizes greater than 1Mb in length [8]. Such findings are not necessarily surprising as, in theory, the impact of an inversion is primarily related with its breakpoints location [8] and if no gene is disrupted, even large inversions may be neutral and, thus, spread within and between populations through stochastic processes.

However, in the absence of a robust high-throughput method to genotype balanced rearrangements, much uncertainty remains regarding the incidence of inversions in humans, how they are distributed throughout populations and their frequency as polymorphic variants. 

## HUMAN POLYMORPHIC INVERSIONS – WORLDWIDE DISTRIBUTION AND EVOLUTIONARY TRAJECTORIES

Aside from a small number of examples that come from indirect studies focusing on human diseases [19, 61, 62], only a couple of inversions have been extensively characterized at the population level [21-24]. Namely, (i) the 8p23.1 inversion that spans a 4.5 Mb region and is considered the largest polymorphic inversion known in the human genome [24], and (ii) the smaller but still very large 900 Kb inversion at 17q21.31 which attains relatively high frequencies in several European populations.

### The 8p23.1 Inversion (*8p23.1-inv*)

Initial studies [19, 63] have made clear that this particular segment presents a very complex genomic architecture mainly due to the two large blocks of segmental duplications (SDs) it contains. Although considered a neutral polymorphism [24], it has been repeatedly argued [64] that, due to the presence of these highly identical structures, subsequent rearrangements via non allelic homologous recombination (NAHR – i.e. a mechanism of illegitimate recombination between sequences of high identity) can cause syndromic phenotypes (e.g. microdeletion syndromes) in the offspring of heterozygous mothers. However, the exact molecular mechanisms leading to disease phenotypes remain to be elucidated (but see below).

Another important aspect of the *8p23.1-inv* is the number of genes encompassed. The region contains at least 50 genes [63], among which the BLK - *B lymphocyte kinase* - gene that has been associated with systemic lupus erythematosus (SLE), rheumatoid arthritis (RA) and other autoimmune diseases [65]. Interestingly, it has been suggested that the risk alleles are specific to the non-inverted configuration [24]. 

In order to characterize its worldwide distribution, Salm *et al.* have recently applied an innovative approach to diploid SNP-genotype data [24]. Taking into consideration the limitations of most SNP-based tagging methods to identify inversions, as we noted above, the authors have designed a new and powerful multidimensional scaling (MDS) algorithm called PFIDO (Phase-Free Inversion Detection Operator) to efficiently categorize almost 2000 individuals from 56 populations by inversion status. 

According to their results, this inversion polymorphism displays a worldwide clinal distribution with frequencies reaching 79% in a Mozabite sample (Algeria), 63% in an Italian sample and 25% in a “Manchu” sample (North-East Asia), which, the authors claimed would be consistent with demographic models of early human expansions out of Africa. However, since no single SNP was perfectly correlated with the inversion status, the 8p23.1 inversion may not act as an absolute recombination barrier and low levels of gene flow may have occurred throughout its evolution. This is not necessarily surprising given the size of the inversion, which may allow for some double cross-over events. 

Based on these results, the authors concluded that the *8p23-inv* appears to have evolved neutrally (or under very weak selective pressure) in humans. Moreover, given the correlation between the genetic substructure and the inversion status, they suggested that recurrent events were also infrequent across this region in the *Homo* lineage.

### The 17q21.31 Inversion (*17q21.31-inv*)

Another relatively common inversion polymorphism that became the focus of intense research in the last years is located at 17q21.31. In contrast to the *8p23-inv*, early studies suggested [21] that the 900 kb inversion polymorphism is undergoing selection in Europeans. After analyzing more than 29,000 Icelandic individuals, Stefansson *et al.* [21] observed that females carrying either one or two copies had more children, and, applying coalescent simulations, concluded that positive selection is likely acting on the rearrangement. 

More recently, Zody *et al.* [22] analyzed the evolutionary history of the same inverted region, using data from several non-human primates. According to their results, this particular segment was prone to multiple recurrent events throughout primate evolution, which contributed to the complex duplicated architecture of the region. Moreover, they highlighted the emergence of directly oriented blocks of segmental duplications (SDs) in the human H2 haplotype (inversion-associated haplotype). SDs can act as substrates of non-allelic homologous recombination (NAHR) that can result in microdeletions and microduplications events, often associated with disease [22, 66, 67]. On this basis, Zody *et al.* [22] proposed that, due to the negative selection against the H2 haplotype, the H1 “chromosome” rose to high frequencies in humans. However, the high frequency of the H2 chromosome in some European populations (between 5 and 35%) was explained by founder effects during the peopling of Europe following the Out-of-Africa human colonization of the continent.

Similar demographic interpretations were subsequently given by Donnelly *et al.* [23] after analyzing a more detailed global distribution of the 17q21.31 haplotypes, using SNPs and short tandem repeats (STRs) polymorphisms. They found low frequencies of the H2 haplotype in most of the 63 non-European populations. Based on these observations, their model favored a complete fixation of the H1 haplotype followed by a *de novo* occurrence in the *Homo* line, hence explaining its patchy distribution. Donnelly *et al.* [23] also concluded that the Neolithic transition, rather than the first out of Africa wave, might be responsible for its present-day distribution in Europe.

Interestingly, two new and independent studies have focused on the duplicated architecture of the 17q21.31 region to further investigate its evolutionary trajectory [68, 69]. Using NGS data from more than 800 individuals and applying a strategy that combined BAC-based assemblies, read depth-base copy number estimates, BAC pool sequencing and FISH, Steinberg *et al.* [68] have identified distinct copy number polymorphisms (CNPs), including a short (CNP155) and long duplication (CNP205) exclusively associated with the H2 and H1 haplotypes, respectively. On the basis of these architectural differences, the authors were able to define four main structural haplotypes classified according to the inversion status and copy-number status. Furthermore, the frequency of the 17q21.31-inv in the African continent was reassessed by surveying a large collection of new population samples from different sources (e.g. 1000Genomes). Remarkably, it was reported that the different inversion-associated haplotypes (namely H2’ and H2D) were segregating at fairly high frequencies (e.g. 7% in Maasai population) in several African ancestry groups, in opposition to earlier observations [23]. 

In light of these new results, Steinberg *et al.* [68] proposed a new model where an ancestral H2 haplotype arose in central or eastern Africa and spread to southern regions before the emergence of anatomically modern humans. Approximately 2.3 Million years ago the region (re-)inverted back to the direct orientation and the resulting genomic configuration (H1) spread throughout the *Homo *lineage becoming the predominant haplotype. The authors also note that the complex duplicated architecture of extant haplotypes (H2D and H1D) represents younger evolutionary events, as the duplications in the two major clades (H1 and H2) have occurred independently. 

Another important conclusion from this study was finding that only one haplotype (H2D) predisposes to the syndromic 17q21.31 microdeletion, via NAHR. This configuration is characterized by the presence of directly oriented homologous SDs flanking the disease-critical region and it is associated with a duplication of the *KANSL1* locus. Intriguingly, this chromosomal variant appears to be enriched in some European populations, with frequencies reaching 25%, and with virtually no genetic variation between carriers. 

Similar conclusions were reached in a parallel study by Boettger *et al.* [69], where two duplications of the *KANSL1* locus, one in each genomic background (H1 and H2), have also been reported. According to the authors, these architectural changes lead to a similar alteration at the molecular level creating a new transcript of the *KANSL* gene which may have an impact on female fertility, as demonstrated in a Drosophila mutant. [70], strengthening the initial idea of selection [21]. 

In summary, the (i) contradictory hypotheses raised to explain the high genetic divergence observed between the inverted and non-inverted configuration in modern humans, and (ii) the conflicting scenarios proposed to explain the expansion of inversion-carrying haplotype across populations, highlight two very important features of genetic data. First, complex spatial phenomena (e.g. human demographic expansions, contractions, and admixture events) can produce selection-like signatures in the genome [71, 72]. And secondly, species-specific characteristics, such as migration rates, population size, etc., are crucial when modeling genetic data. It is well known that human populations have gone through massive changes in size and distribution in the past, including expansions, bottlenecks, and admixture events, which resulted in distinct genetic diversity patterns among populations. However, quantifying the contribution of past events to the genetic pool of present-day populations remains a difficult task [73] in which new modeling approaches are needed.

Due to the complexity of the 17q21.31 region, the evolutionary history of this inversion remains a debated issue [68]. Although one cannot rule out the possibility of selection (nor a possible contribution of the *Homo neanderthalensis* [74]), it is quite likely that different demographic histories could produce the same patterns of variation with or without selection. Identifying the scenarios that best explain these patterns is a challenge that may be overcome with some recent advances in population genetics inference.

### Simulation and Inferential Tools

One important question is whether there is an appropriate statistical framework which would allow us to choose among a set of currently proposed scenarios the most appropriate. Recent advances in population genetics modeling suggest that it may be possible thanks to improved simulation programs and to Approximate Bayesian Computation (ABC), which may provide part of the answer. In a few words, the ABC framework relies on the use of very large numbers of simulations under one or several models. The observed (or real) genetic data are summarized by several summary statistics such as the number of alleles or the expected heterozygosity. The simulated data are also summarized and compared to the observed data. The scenarios or parameter values that produce simulated data that are closest to the observed data are then considered to be the most likely ([75, 76] for a review). The ABC methodology relies on the ability to simulate genetic data very efficiently and rapidly, which was made possible thanks to the development of the coalescent theory [77]. In the last ten years the ABC framework has gained momentum and has been widely applied. It is the focus of intense research [78-81] which suggests that it is a very flexible approach to model choice and parameter estimation. In the case of genomic data and inversions, one of the main constraints is the limitation in terms of simulating tools. While simulating large numbers of loci under the coalescent is relatively straightforward [76], even at a genome-wide scale [82], the simulation of inversions has unfortunately received little attention with few exceptions [83].

To our knowledge, invertFREGENE [83] is the first (and probably the only) software allowing the introduction of a single inversion polymorphism of specific length into a population. The authors ingeniously modified a version of a previously published software [84] to incorporate the possibility of modeling neutral inversion rearrangements under a finite sites mutation model. 

The invertFREGENE software provides the possibility of simulating very large inversions, and to account for complex demographic scenarios to study the fate of inversions. Several features like the incorporation of population substructure, instantaneous expansions and contractions, are also allowed. However, there are several limitations which make it difficult for statistical inference. Indeed, invertFREGENE allows the simulation of inversions by specifying a “target” frequency (for instance the observed frequency today) but, since the number of simulations that actually took place in order to reach this target frequency is not kept, it is difficult to identify the parameter values most likely to produce the observed data. In other words, each run only gives the output for one successful inversion that reached the given target frequency. However, given that the code is freely available it should be possible to modify it so as to circumvent this limitation.

By using its core simulation engine, one could in principle develop an ABC approach that would allow us to identify models of recent human evolution with and without selection that best explain the current distribution of inversions in human populations. Recent simulation work by Li and Jakobsson [85] has for instance shown that the use of between several hundreds and a couple of thousands of SNPs, provides major improvements in the estimation of parameters. They did not explore the issue of model choice but other studies have done it with smaller number of loci [81, 86] For instance Fagundes *et al.* [86], were able to identify which model of human evolution was best supported using only 50 independent DNA sequences. With the arrival of genomic data, one could potentially determine how different regions of the genome are best explained by models with or without selection. Inverted regions could easily be typed for hundreds of SNPs and their demographic history compared to that of other regions. The general ABC framework has its limits. For instance, using forward-in-time simulators, such as invertFREGENE, could prove computationally very demanding. However, it is currently one of the most flexible and powerful approaches to explore the properties of genomic data, including inversions.

## HUMAN INVERSIONS - OVERLOOKED ISSUES AND FUTURE PERSPECTIVES

### Disease Associated Inversions

That the inversion of a DNA segment could interfere with gene function by disrupting its reading frame or rearranging the position of promoters, enhancers and other regulatory elements, should not be surprising. However, with the exception of a recurrent inversion located on the X chromosome [87], most human inversions do not appear to be directly linked to disease.

More often inversion rearrangements are associated with complex genomic disorders, as recently reviewed in [8]. In fact, due to the characteristic duplicated architecture of inversion breakpoints, they apparently increase the probability of disease through the occurrence of unbalanced rearrangements in the offspring [8, 22, 66, 67].

As seen above, if the duplicated copies present the same orientation they may lead to deletion or duplication events, as a result of NAHR [66, 67]. Theoretically, both events are expected to occur in equal proportions [88], however duplication-associated syndromes are rarely reported [89]. 

Several interpretations can be given for this observation. For instance, it has been suggested that, due to the phenotype variability observed in patients with NAHR-mediated duplications [88, 89], mild effects tend to be underdiagnosed, generating an ascertainment bias in evaluating the frequency of duplication-associated disorders.

Alternatively, one might also hypothesize that, depending on the size and the number of genes located on the duplicated region, extremely severe outcomes (i.e. abortion) might also result (e.g. as a consequence of gene dosage) from such events. However, our current interpretations rely on estimates of transmitted chromosomes, potentially generating a bias against negatively selected gametes. As a consequence, these suggestions remain merely speculative. 

Lastly, the mechanistic details by which inversions contribute to complex genomic disorders are still unclear. Even with emerging technologies allowing the characterization of inversion breakpoints, SDs vary extensively in copy number [4] and understanding how these polymorphic features can simultaneously contribute (i) as a source of genetic variation, and (ii) to the establishment of human disorders, remains an important challenge to human genetics research. 

### Inversion Hotspots

From an evolutionary perspective, the presence of almost identical duplicated sequences in inversion breakpoints is also intriguing. Consider, for instance, the whole-genome comparative study by Murphy *et al.* [90] where the genome organization of 8 mammalian species was analyzed in order to identify patterns of chromosome evolution. Using homologous synteny blocks (HSBs) they have identified several regions of chromosome breakage that apparently have been reused throughout evolution (i.e. independent breaks occurring at the same chromosomal sites). Interestingly, the authors have also observed that most of primate-specific breaks involve inversions that have been generated via NAHR between duplicated HSBs.

Further support was later provided by Caceres *et al.* [91] who identified another example of long-term breakpoint reuse throughout mammalian evolution in a genomic segment containing a polymorphic inversion on the human X chromosome. By sequence comparison between 28 placental mammals, the authors have suggested that at least 10 independent recurrent events must be considered to accommodate the present-day genomic structures observed in different species. In addition, recurrent events within multiple primate lineages have also been proposed for the 17q21.31 region [22]. 

Overall, these results appear to suggest that some genomic locations might exhibit greater rearrangement activity than others. One interesting possibility is that some regions represent conserved inversion hotspots that could have been maintained due to important functional or regulatory properties associated with the duplications [91]. Indeed, after analyzing a specific class of duplicated structures, defined as inverted repeats (IRs), Warburton *et al.* [92] hypothesized that their maintenance during primate evolution could be linked to important regulatory mechanisms controlling deleterious gene expression on sex-chromosomes. 

In conclusion, future work is still needed in order to determine the distribution of these apparently non-randomly distributed break sites, as studies analyzing at depth the population genetics of inversions are scarce in the literature. 

### Inversions and Recombination Rate

Many authors have also overlooked the effect of chromosomal inversions on the overall recombination rate, despite the vital role of crossing over during meiosis for proper chromosome segregation [93, 94].

In humans, as in many other organisms [95, 96], recombination is affected by several genomic features, such as location (e.g. lower rates near centromeres and higher near telomeres), and gene density (but see [97] for a more detailed review). Interestingly, it has also been shown that most recombination events (approximately 80%) are concentrated in small genomic regions of 1-2 kb, known as recombination hotspots [98-100].

The PRDM9 gene was recently described [101, 102] as a major regulator of human recombination hotspots, with allelic variants of this gene influencing the differential usage of recombination hotspots. However, one might hypothesize that if an inversion happens to encompass an active hotspot, recombination will likely become inhibited in that particular region, disturbing the overall recombination rate by possibly de-localizing crossing-over events to different locations. For instance, it has been argued that, in *Drosophila* species, inversions significantly increase the recombination rate throughout the rest of the genome [94]. Interestingly, it has also been consistently reported that polymorphisms on the H2 (inverted) haplotype in 17q21.31 are associated with an increase of the genome-wide recombination rate in heterozygous females [21, 103]. Have these inversions trapped specific variants of more active recombination hotspot determinants? That is an intriguing possibility; however, the recombination machinery might be extremely different between these species, since no recombination hotspots were ever reported in *Drosophila* [97]. 

On an evolutionary time-scale, inversions may lead to new stabilizing points of the map of recombination events within the affected chromosome, as has been recurrently observed in the establishment of dimorphic sex chromosomes of mammals and other distantly related vertebrate taxa [104-106] as well as in plants [107]. In fact, incipient heteromorphic sex chromosomes (Y and Z chromosomes) often differentiate via the accumulation of inversion rearrangements that prevent recombination over increasingly large regions with their homologues. Nevertheless, recombination and successful disjunction are maintained and therefore the recombination machinery may be more labile than would be expect *a priori*.

Moreover, since current estimates suggest that approximately 25,000 putative hotspots exist in the human genome [100] understanding how inversion rearrangements might affect or contribute to differential hotspot usage will be a challenging task.

## CONCLUSIONS

Given the increased interest on chromosomal rearrangements, scientists are now beginning to recognize inversions as important players shaping genetic variation. Over the last decade, fundamental questions began to emerge focusing on their molecular properties [66, 67], on the mechanisms responsible for their origin [108], on their evolutionary significance [1, 47, 109] and on their role in speciation [14, 47, 48, 50-52].

In humans, extensive sequencing efforts have revealed a somewhat surprising abundance of inversions segregating as polymorphisms [29]. This observation is in sharp contrast with previous expectations that suggested a direct impact of inversions on fertility [109]. However, as seen above, it is evident that such impact might be influenced by a combination of multiple processes [110, 111]. 

As genomic information continues to accumulate in publicly available databases, new *in silico* approaches combined with evidences of human demographic history – based on archaeological and linguistic theories - might prove useful when exploring the role of inversion polymorphisms as evolutionary significant elements. Nevertheless, genetic data should be used with extreme caution as different plausible scenarios might fit the observed patterns of present day diversity [72, 73].

In our opinion, due to its flexibility, robustness and efficiency, ABC (or other genomic inference) strategies should be considered in future studies, as these approaches allow us to quantify the relative contribution of ancient and recent factors, including selection, in shaping the genetic structure of present-day populations. Even if ABC modeling only represents an approximation, they surely constitute a promising statistical inferential framework to reconstruct important aspects of the evolutionary history of populations. 

In addition, another limitation in most inversion-based evolutionary studies is that most authors only consider the evolutionary effects of a single genomic inversion. However, several inversions might operate simultaneously on an individuals’ genome, and, no matter how accurate the methodology used, some confounding variables might create an apparent association or mask a real one. Fortunately, we are at a stage where comparative data might enable us to answer some of these questions.

In conclusion, future studies merging evolutionary and molecular perspectives will allow us to understand the implications of this specific type of structural variability to complex diseases, and how selective factors could have influenced their evolution.
